# Effects of an untreated medial meniscal ramp lesion on histological deterioration findings of the medial meniscus: A study in a porcine anterior cruciate ligament reconstruction model

**DOI:** 10.1002/jeo2.70027

**Published:** 2024-09-27

**Authors:** Akihiro Saitsu, Tsuneari Takahashi, Hibiki Kakiage, Kazuhisa Hatayama, Tatsuya Kubo, Yuta Matsumoto, Katsushi Takeshita

**Affiliations:** ^1^ Department of Orthopaedics, School of Medicine Jichi Medical University Shimotsuke Japan; ^2^ Medical Education Center, R & D Division of Career Education for Medical Professionals Jichi Medical University Shimotsuke Japan; ^3^ Department of Orthopaedic Surgery, School of Medicine Gunma University Maebashi Japan; ^4^ Department of Orthopaedic Surgery Japan Community Health Care Organization Gunma Central Hospital Maebashi Japan; ^5^ Department of Orthopedic Surgery Shin Oyama City Hospital Oyama Japan; ^6^ Department of Orthopedic Surgery Nasu Minami Hospital Karasuyama Japan

**Keywords:** anterior cruciate ligament reconstruction, biomechanical study, histological evaluation, medial meniscal ramp lesion, porcine model

## Abstract

**Purpose:**

To evaluate the effect of untreated medial meniscal ramp lesions (MMRLs) on the tendon graft after anterior cruciate ligament (ACL) reconstruction and histological findings of medial meniscus (MM) in a porcine a model.

**Methods:**

A total of 17 pigs were divided into two groups: (1) the untreated MMRL group (UM group, *n* = 9) and (2) intact MM group (*n* = 8) and euthanized 12 weeks after surgery. The specimens were then tested cyclically and loaded to failure. Side‐to‐side differences (SSDs) in translation under cyclic loading and structural properties were analyzed. Histological evaluation of the MM was also performed.

**Results:**

No significant differences in the SSD in translation during the cyclic testing (UM group, 0.3 ± 0.4 mm; intact MM group, 0.1 ± 1.4 mm), upper yield load (UM group, 476.3 ± 399.9 N; intact MM group, 643.2 ± 302.9 N), maximum load (UM group, 539.5 ± 265.8 N; intact MM group, 705.8 ± 282.6 N), linear stiffness (UM group, 63.5 ± 39.0 N/mm; intact MM group, 73.7 ± 60.1 N/mm) and elongation at failure (UM group, −4.6 ± 16.3 mm; intact MM group, 2.3 ± 6.6 mm) were observed. However, the UM group had significantly worse Modified Mankin's histological grading scores (1.8 ± 0.4 [1–2] vs. 0 ± 0 [0]; *p* < 0.001) and Modified Copenhaver classification scores (6.6 ± 2.4 [2–9] vs. 0.7 ± 1.1 [0–3]; *p* < 0.001) than did the intact MM group.

**Conclusion:**

Untreated MMRLs showed postoperative histological deterioration.

**Level of Evidence:**

Level IV.

**Abbreviations**
ACL: anterior cruciate ligament
FAT: femur–anterior cruciate ligament–tibia
FGT: femur–graft–tibia
MM: medial meniscus
MMRL: medial meniscus ramp lesion
SSD: side‐to‐side difference

## INTRODUCTION

Although the ACL acts as the primary restraint against anterior tibial translation, the medial meniscus (MM) does play an important role as a secondary restraint. Recently, MM ramp lesions (MMRLs), defined as a peripheral meniscal detachment of the posterior MM [13] concomitant with ACL injury, have been attracting some attention. The overall prevalence of MMRLs in patients with ACL injuries was high up to 39.5% [4] and male sex, younger age, lateral meniscal injury and percentage of ACL remnant is reported as significant risk factors for MMRLs concomitant with primary ACL injury [12]. Accordingly, studies have reported that MMRL repair is biomechanically important for restoring the kinematic properties of the knee and preventing increased ACL graft strain [19]. On the other hand, no studies have so far demonstrated a higher failure rate for ACL reconstruction with MMRL, a conflicting result for MMRL repair, as several studies suggest that repair is not necessary, especially if the tear is stable [1, 2]. There is still no clinical evidence to support systematic repair of all MMRLs encountered during ACL reconstruction. Biomechanical cadaveric studies have inherent limitations because they do not take into account the biological aspects. In addition, little is known about in vivo healing and size progression of MMRLs when left in the ACLR [3]. The current study therefore aimed to evaluate whether untreated MMRLs affect the postoperative biomechanical properties and histological remodelling of the tendon graft and whether the procedure affects the laxity of the graft under cyclic loading, the structural properties of the tendon graft itself, and histological changes in the MM in a porcine model of ACL reconstruction. Accordingly, we hypothesized that untreated MMRLs would worsen the postoperative biomechanical properties and histological remodelling of tendon grafts, as well as worsen postoperative MM histologic scores in a porcine model of ACL reconstruction.

## MATERIAL AND METHODS

This study used 2‐month‐old male castrated pigs (Sanesu Bleeding; mean weight 25.7 kg, range 19.0–32.0 kg). All animal experiments were conducted in accordance with the rules of our institution's Animal Care and Use Committee, and each of the 18 pigs was randomly assigned to an ACL reconstruction group with MMRL (untreated MMRL group [UM group], *n* = 9) or without MMRL (intact MM group, *n* = 9) using a random number table. All left knees were assigned to sham surgery to determine the side‐to‐side difference (SSD).

### Surgical procedures

**Figure 1:**
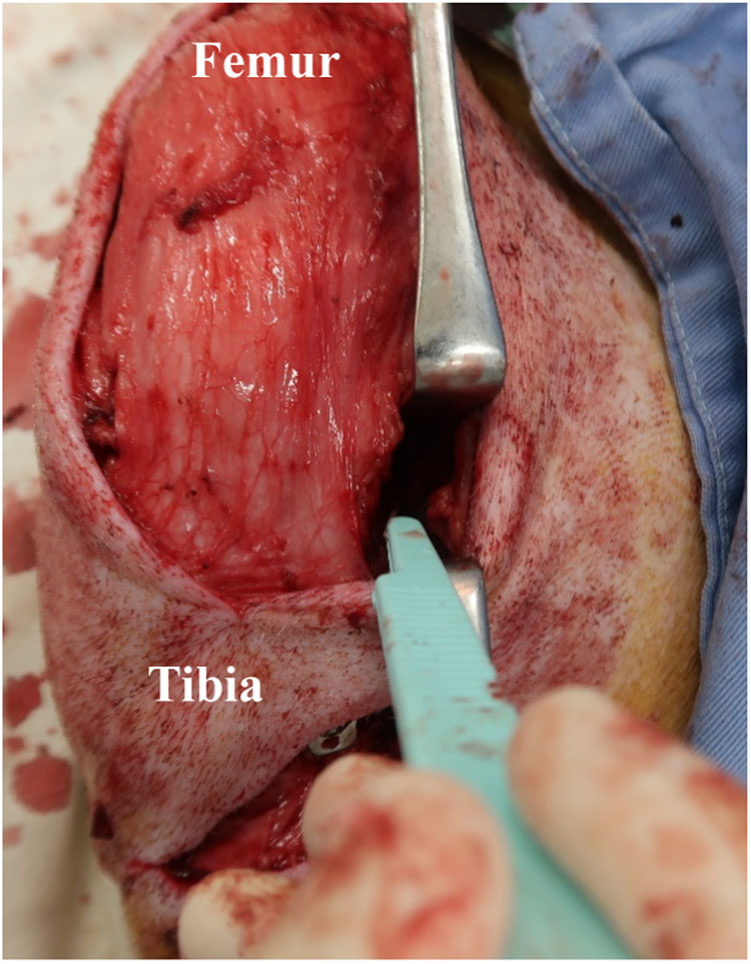
Incision at the interface between the posterior horn of the medial meniscus and the posterior capsule. The interface between the posterior horn of the medial meniscus and the posterior capsule was incised beginning adjacent to the course of the posterior cruciate ligament and continuing 10‐mm medially. The vertical orientation of the scalpel was maintained and continued inferior to the tibial plateau throughout the incision at the junction of the posterior aspect of the MM and the posterior capsule, creating a MMRL that spanned the entire meniscus–capsule junction. MM, medial meniscus; MMRL, medial meniscal ramp lesions.

All surgeries were performed by an experienced knee surgeon (T. T.). Under intubated general anesthesia and asepsis, a skin incision was made longitudinally across the midline of the right knee. The semitendinosus tendon was then harvested from the distal portion of the incision. Thereafter, the grafted tendon was harvested at a width of 6 mm and trimmed to a length of 50 mm when folded over to produce a grafted tendon. The tendon was connected using a continuous loop of RIGIDLOOP (Mitek Sports Medicine). The tibial ends of the tendon graft were sutured using two No. 2 FiberWire sutures (Arthrex). After performing a lateral parapatellar arthrotomy, the ACL was excised. A femoral–tibial bone tunnel was created at the centre of the ACL attachment site. The femoral tunnel was created on the guide pin from the inside out using a 4.5‐mm cannulated drill, followed by a 20‐mm drill with a 6‐mm diameter. The tibial tunnel was inserted over the guide pin from the inside out using a 4.5‐mm cannulated drill, followed by a 6‐mm cannulated drill. The tendon graft was introduced into the joint cavity through the tibial tunnel and placed in the femoral socket. After fixing the femoral side of the graft using RIGIDLOOP, an initial tension of 40 N was applied to the graft, followed by fixation of the tibial side of the graft using a Double Spike Plate (DSP; Smith & Nephew Endoscopy) and cancellous bone screws at 60° of knee joint flexion [8, 20]. After graft fixation, a 10‐mm MMRL was created in the UM group using the posterior medial open approach based on the method used in previous studies [13, 16, 17, 19]. The incision at the junction of the posterior aspect of the MM and the posterior capsule was created starting adjacent to the posterior cruciate ligament and continuing medially for 10 mm (Figure 1). The vertical orientation of the scalpel was maintained and continued inferior to the tibial plateau throughout the incision at the junction of the posterior aspect of the MM and the posterior capsule, creating a MMRL that spanned the entire meniscus–capsule junction [17].

### Sham surgery

For the sham surgery, both the UM and intact MM groups underwent a midline longitudinal skin incision and immediate wound closure on the left knee of each animal.

### Postoperative management

**Figure 2:**
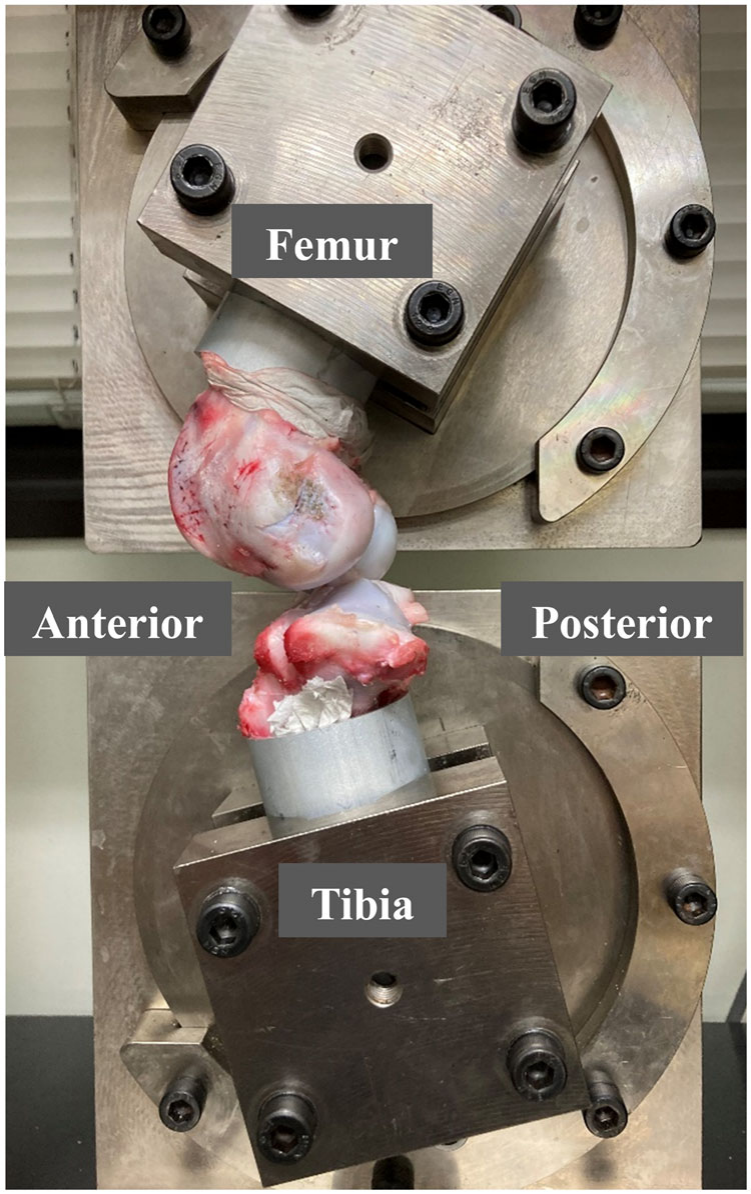
Knee specimens mounted on a tensile tester using a set of specially designed grips. The femur and tibia were potted separately into aluminum tubes with cement. Knee specimens were kept moist during experiment and mounted on a tensile tester using a set of specially designed grips, after which the tibia was flexed to 45° relative to the femur.

After surgery, the animals were returned to their cages (2 × 3 × 2 m) and allowed full weight‐bearing without movement restriction [8]. All animals were observed once to twice per week to monitor for the occurrence of protective limping or pus discharge. All animals exhibited normal gait within 2 weeks after surgery. Given that one animal in the intact MM group died during the follow‐up period, 17 animals were eventually euthanized at 12 weeks after surgery in accordance with the Animal Care and Use Committee regulations. We then evaluated the effects of untreated MMRLs on autologous tendon remodelling at the 12‐week postoperative timepoint, following available evidence regarding the timing at which the structural properties of autologous transplanted tendons become weakened in large animal ACL reconstruction models [10]. The mean weight of the animals at euthanasia was 45.4 kg (range, 38.0–51.6 kg). At the time of euthanasia, a gross evaluation of the operated knee joint was performed to document the appearance of the graft and secondary changes, such as synovitis, articular cartilage lesions and meniscus tears. Knee joint specimens were retrieved immediately after euthanasia. After the femur and tibia were cut 13 cm from the joint line, a sharp scalpel was used to remove all surrounding muscles, the patella, the patellar tendon and other ligaments to avoid damage to the meniscus. The fibula was then resected distal to the lateral collateral ligament attachment site, after which the DSP and cancellous bone screws were removed from all right knees. With the removal of the DSP, the tendon graft was attached to the tibia only at the tendon–bone junction in all right knees. The femur and tibia were potted separately into aluminum tubes with cement (Figure 2) [8, 11, 16].

### Biomechanical evaluations

#### Drawer testing

Each specimen was kept moist using a saline spray throughout the procedure. The axial translation of the knee was measured under a drawer force using a previously reported testing condition [8, 11]. Knee specimens were mounted on a tensile tester (Tensilon RTG 1250; Orientec) with a set of specially designed grips. The tibia was flexed to 45° relative to the femur (Figure 2) [8, 11, 16]. Before testing, the specimens were preconditioned with a static preload of 5 N for 30 s, followed by 20 cycles of loading between 0 and 40 N with a crosshead speed of 100 mm/min to simulate the tibial anterior drawer setting. The axial translations after the 20th cycle were then measured using the Tensilon Advanced Controller for Testing software (Orientec). These measurement conditions were the same as those used in a previous biomechanical study using porcine models [8, 11].

#### Structural properties of the femur–graft–tibia (FGT) complex

**Figure 3:**
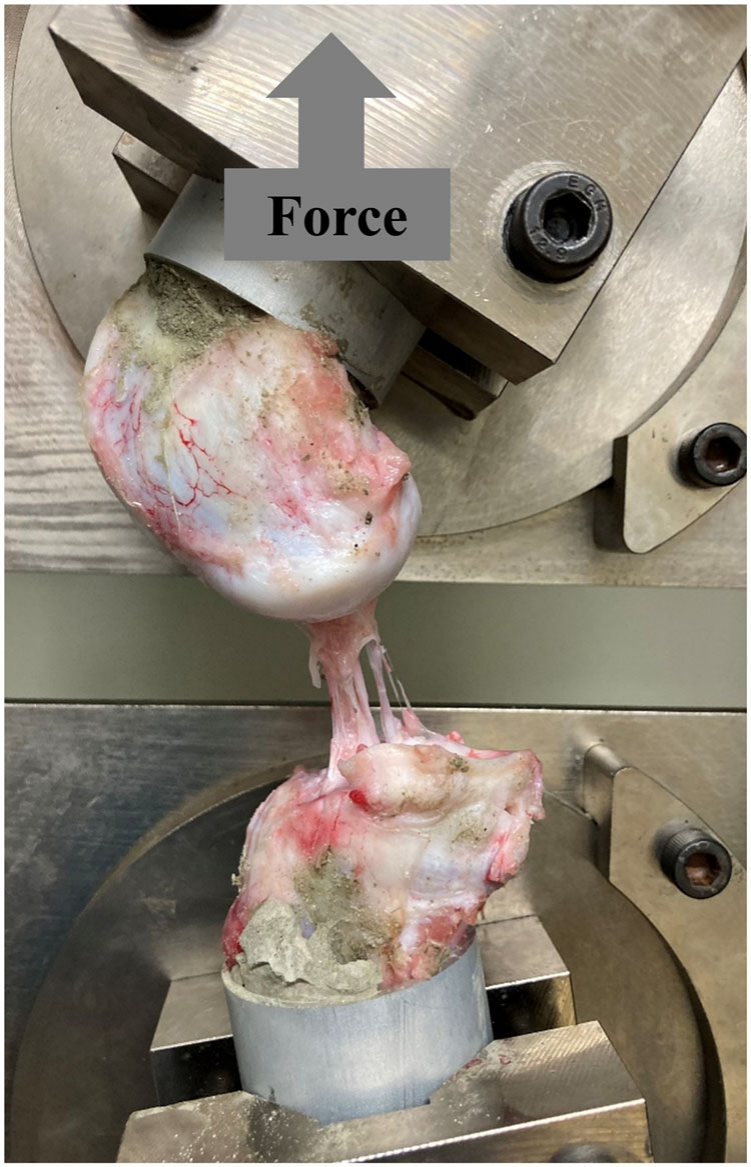
Biomechanical evaluation of the femur–graft–tibia (FGT) complex. The FGT complex was mounted on a tensile tester using a set of specially designed grips. A tensile load was then applied to the grafted tendon parallel to its long axis. Cyclic testing was performed initially to simulate the tibial anterior drawer setting, followed by tensile testing. Failure modes were recorded. The structural properties (upper yield load, maximum load, linear stiffness and elongation at failure) of the complex were determined through software calculations.

After cyclic testing, all menisci were carefully removed except for the reconstructed graft or native ACL. The prepared FGT or femur–native ACL–tibia (FAT) complex specimens were mounted on the tensile tester using a set of specially designed grips. Thereafter, the tibia was flexed to 45° relative to the femur to apply a tensile load to the grafted tendon parallel to the long axis (Figure 3). Before the tensile test, the specimens were preconditioned with a static preload of 5 N for 10 min, followed by 10 cycles of loading and unloading (3% strain) at 20 mm/min. Afterward, each specimen was loaded to failure at 50 mm/min. These conditions had been used in previous studies with large animal models [8, 11, 20]. Failure modes were recorded. A load–elongation curve was created using Tensilon Advanced Controller for Testing software. The structural properties (upper yield load, maximum load, linear stiffness and elongation at failure) of the FGT or FAT complex were determined through software calculations.

### Histological evaluations

Immediately after biomechanical examinations, the MM was harvested from the knee and fixed using a 10% buffered formalin solution (pH = 7.4) for 24 h at 4°C, followed by decalcification with ethylenediaminetetraacetic acid for 7 days. After embedding in paraffin, 5‐µm‐thick longitudinal sections were cut in the sagittal plane along the longest axis of the graft. Each section was mounted onto a glass slide coated with 0.01% poly‐L‐lysine. The sections were dried overnight at 37°C and dewaxed in xylene. The sections were then rehydrated with distilled water, soaked in phosphate‐buffered saline (pH = 7.4) and stained with hematoxylin and eosin for histomorphological observation. The sections were evaluated using light microscopy. The extent of meniscal degeneration was evaluated using the Modified Mankin's histological grading score and Modified Copenhaver classification score (Tables 1 and 2) [15]. All specimens were assessed independently by two board certified orthopedic surgeons (T. T. and H. K.).

**Table 1:** Modified Mankin's score for meniscus.

*Collagen structure*
0 Normal
1 Slight disturbance
2 Moderate disturbance
3 Severe disturbance/mucoid substances
*Cellular abnormalities*
0 Normal
1 Hypercellularity
2 Cloning tendency
3 Hypocellularity
*Proteoglycan content*
0 Normal
1 Slight reduction
2 Moderate reduction
3 Severe reduction
4 No dye noted

**Table 2:** Modified Copenhaver classification.

Grade	Criteria
0	Homogeneous eosinophilic staining collagen reinforced ground substance with normal chondrocytes
1	Discrete foci of mucinous, hyaline or myxoid degeneration and reduction of the chondrocyte concentration
2	Bands of mucinous degeneration bordering hypocellular regions of the meniscus without presence of a distinct cleavage plane
3	Mucinous degeneration with fibrocartilaginous separation

### Statistical analyses

Continuous data were presented as means and standard deviations and compared using Student's *t* test. Ordinal data were presented as medians and ranges and compared using the Mann–Whitney *U* test. Categorical data were compared using Fisher's exact tests. A priori power analysis was conducted based on the results of the study by Takahashi and colleagues, who previously reported the mean anterior tibial translation values for the remnant preserved group (9.3 ± 2.1 mm) and remnant removed group (5.4 ± 1.7 mm) in a large animal model of ACL reconstruction [20]. Although a previous study on ligament augmentation in the same animal model used in the current study would have been desirable, no such study is available. We determined that eight specimens per group would provide a power of 80% to detect a difference (*α* < 0.05) in the mean axial translation. The inter‐rater reliability of the Modified Mankin's histological grading score and Modified Copenhaver classification score were assessed using Fleiss's *κ* statistics. All statistical analyses were performed using EZR software [9], with *p* < 0.05 indicating statistical significance.

## RESULTS

### Gross observations in the knee joint

After the surgery, no signs of infection or arthrofibrosis were observed. All tendon grafts were intact and covered with synovial tissues. No obvious degenerative changes on the articular cartilage were observed at the time of euthanasia. Untreated MMRLs in the UM group were covered with granulation tissue.

### Biomechanical evaluations

#### Drawer testing

The SSD in translation during the cyclic testing was 0.3 ± 0.4 mm and 0.1 ± 1.4 mm in the UM and intact MM groups, respectively. No significant difference was observed between the two groups (n.s., Table 3).

**Table 3:** Results of side‐to‐side differences in tensile testing.

Parameters	UM group (*n* = 9)	Intact MM group (*n* = 8)	*p* Value
Displacement, mm	0.3 ± 0.4	0.1 ± 1.4	0.82
Upper yield load, *N*	476.3 ± 399.9	643.2 ± 302.9	0.35
Maximum load, *N*	539.5 ± 265.8	705.8 ± 282.6	0.23
Linear stiffness, *N*/mm	63.5 ± 39.0	73.7 ± 60.1	0.68
Elongation at failure, mm	−4.6 ± 16.3	2.3 ± 6.6	0.29

*Note*: Data are expressed as means ± standard deviation. Student's *t* test was performed.

Abbreviation: UM, untreated medial meniscal ramp lesions.

#### Observation of failure mode on tensile testing

During tensile testing, all tendon grafts in the UM and intact MM groups of the right knee ruptured at the proximal midsubstance, slightly more distal to where the graft looped around the RIGIDLOOP. None of the cases had the grafted tendon pulled out of the tibial bone tunnel. All native ACLs of the left knee had avulsed from the femoral or tibial attachment.

#### Structural properties of the FGT complex

No significant SSD in the upper yield load (UM group, 476.3 ± 399.9 N; intact MM group, 643.2 ± 302.9 N), maximum load (UM group, 539.5 ± 265.8 N; intact MM group, 705.8 ± 282.6 N), linear stiffness (UM group, 63.5 ± 39.0 N/mm; intact MM group, 73.7 ± 60.1 N/mm), and elongation at failure (UM group, −4.6 ± 16.3 mm; intact MM group, 2.3 ± 6.6 mm) was observed between the UM and intact MM groups (n.s., Table 3).

### Histological evaluations

**Figure 4:**
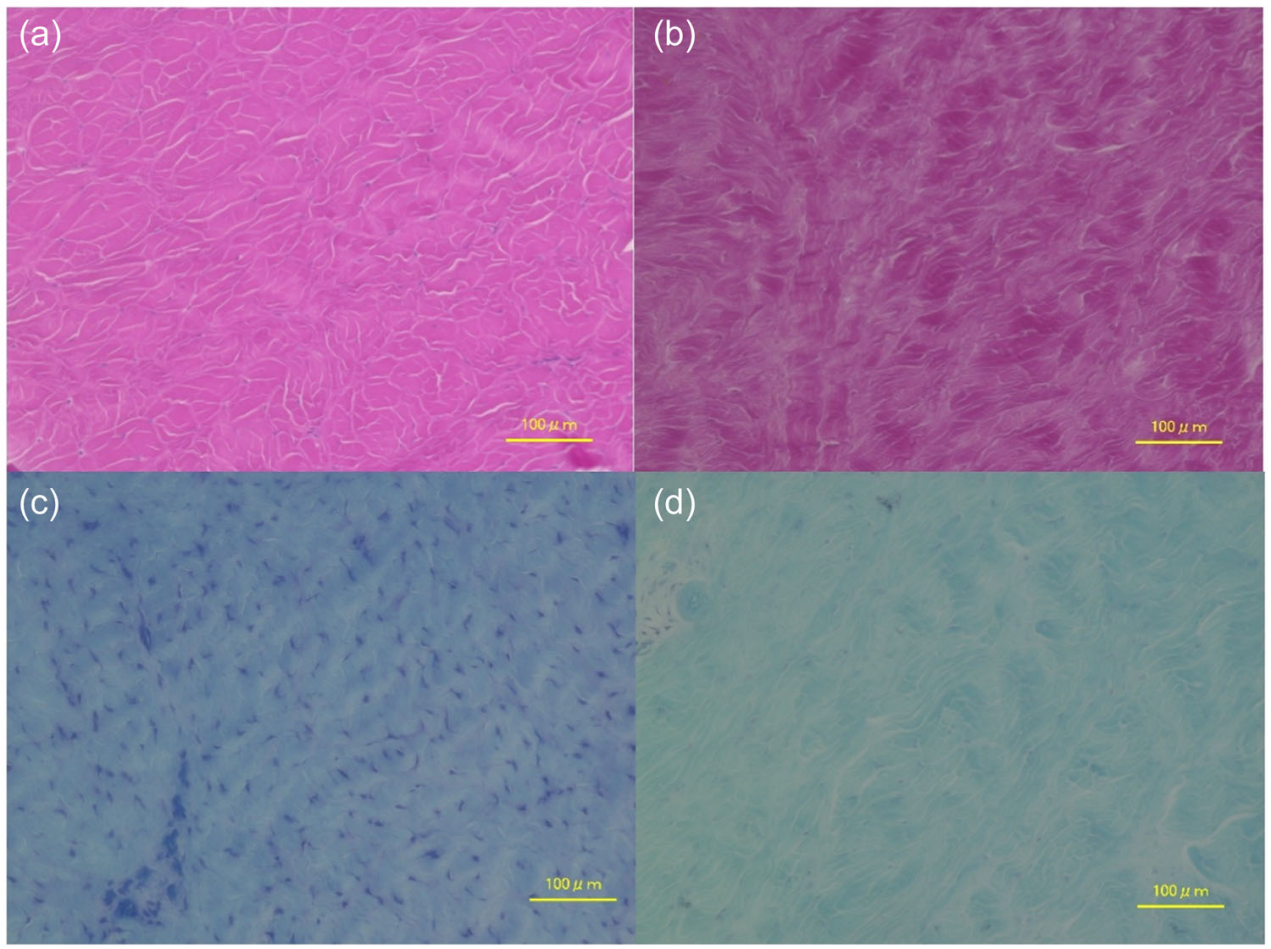
Histological evaluation of the meniscus. (a) Hematoxylin and eosin staining ×200; histologic observations in the intact MM group showing normal cell density, normal proteoglycan staining and regular alignment of collagen fibre fascicles. (b) Hematoxylin and eosin staining ×200; histologic observations in the UM group showing irregular alignment of collagen fibre fascicles. (c) Toluidine blue staining ×200; histologic observations in the intact MM group showing normal proteoglycan content. (d) Toluidine blue staining ×200; histological observations in the UM group showing a reduction in cell density and proteoglycan content. UM, untreated medial meniscal ramp lesions.

Degeneration, which was indicated by changes reflecting a decrease in cell density, minimal proteoglycan staining and irregular alignment of collagen fibre fascicles, was observed more often in the UM group than in the intact MM group (Figure 4).

The UM group showed significantly worse Modified Mankin's histological grading scores (1.8 ± 0.4 [1, 2] vs. 0 ± 0 [0]; *p* < 0.001) and Modified Copenhaver classification scores (6.6 ± 2.4 [2, 3, 4, 5, 6, 7, 8, 9] vs. 0.7 ± 1.1 [0–3]; *p* < 0.001) than did the intact MM group.

## DISCUSSION

The primary findings of the current study revealed that untreated MMRLs did not significantly affect postoperative anterior laxity or the structural properties when ACL reconstruction was performed immediately after MMRL creation. Biomechanical evaluation showed that all tendon grafts in the UM group were torn in the midsubstance, similar to that in the intact MM group. Moreover, no significant SSDs in anterior laxity or structural properties (maximum load, upper yield load, linear stiffness and elongation at failure) had been noted between the two groups. However, histological results showed that the UM group had significantly inferior MM body Modified Mankin histological grading scores and Modified Copenhaver classification scores than did the intact MM group. The results presented in the current study bridges the gap in the literature regarding the efficacy of MMRL repair in conjunction with ACL reconstruction and will be of significant value for future clinical research.

Studies have shown that failure to address MMRLs may increase the forces placed on the ACL graft [19]. Therefore, we hypothesized that untreated MMRLs would worsen the postoperative structural properties of tendon grafts in a porcine model of ACL reconstruction. However, our biomechanical evaluation showed that untreated MMRLs did not significantly affect postoperative anterior laxity or structural properties. One plausible reason for this is our immediate and adequate reconstruction of the ACL graft before MMRL creation. Indeed, DePhillipo and colleagues explained that the ACL acts as the primary stabilizer against anterior translation and that proper ACL reconstruction may prevent significant changes in anterior migration after MMRL creation [6]. The MM, which is firmly attached to the posterior margin of the tibial plateau, acts as a secondary stabilizer against anterior translation and tibial rotation in ACL‐deficient knees. Matsumoto et al., who investigated the ex‐vivo biomechanical efficacy of MMRL repair in ACL reconstruction with respect to the graft protection effect after cyclic loading at time zero, found that simultaneous MMRL repair during ACL reconstruction did not reduce the change in length or anterior translation during cyclic loading when compared to the nontreatment condition. Furthermore, simultaneous MMRL repair during ACL reconstruction did not improve postoperative structural properties [16]. Thus, in the current study, the stability of the ACL reconstruction at 12 weeks after surgery may have been sufficient to avoid overloading the MM, which functions as a secondary stabilizing factor. In addition, we hypothesized that untreated MMRLs would worsen postoperative MM histologic scoring, which had indeed been reflected in our histologic scoring results. Previous histologic studies have shown that MMRL healing is theoretically possible with respect to the vasculature of the menisci fascial synovial junction [7]. Conversely, several clinical studies have reported healing of tears without surgical treatment [14, 21]. In fact, Yagishita and colleagues revealed that a long MMRL may cause poor blood supply, which may require surgical stabilization [21]. Although our findings showed that untreated MMRLs were covered with granulation tissue, the posterior aspect of the MM remained unstable, and inadequate blood supply may have caused histologic deterioration. The overall meniscus repair failure rate remains high up to 19% with a minimum follow‐up of 5 years [18] and it is not yet clear whether surgical repair of MMRLs is necessary in all ACL reconstructions cases [5]. Future studies will be needed to determine whether performing appropriate MMRL repair simultaneously with ACL reconstruction could preserve the histologic status of the MM. Determining the impact of chronic MMRLs on postoperative anterior laxity and structural properties after ACL reconstruction in the chronic phase could also be a matter worth investigating in the future. The current study has several limitations that warrant acknowledgement. First, this study was conducted in young pigs evaluated at an early stage of 12 weeks. Therefore, our results may not be directly applicable to adolescent humans. Moreover, this study did not evaluate the long‐term effects of untreated MMRLs. Second, no biomechanical testing of MMRLs was performed. Third, the results presented herein may vary depending on the surgeon performing the surgery. The results presented in the current study lays the foundation for further clinical studies seeking to investigate the utility of MMRL repair during ACL reconstruction.

## CONCLUSIONS

Untreated MMRLs did not significantly affect anterior knee laxity or structural properties of the grafted tendon 12 weeks after surgery. However, untreated MMRLs showed postoperative histological deterioration.

## AUTHOR CONTRIBUTIONS

The conception and design of the study were done by Tsuneari Takahashi. The acquisition of data was taken care of by Akihiro Saitsu and Tsuneari Takahashi, Hibiki Kakiage, Kasuhisa Hatayama, Tatsuya Kubo and Yuta Matsumoto. Analysis and/or interpretation of data was carried out by Akihiro Saitsu and Tsuneari Takahashi. The drafting of the article was done by Akihiro Saitsu, Tsuneari Takahashi, Hibiki Kakiage and Kasuhisa Hatayama. Revising the article critically for important intellectual content was taken care of by Katsushi Takeshita. All authors have contributed significantly to the study, approved the article and agreed with the submission.

## CONFLICT OF INTEREST STATEMENT

The authors declare no conflict of interest.

## ETHICS STATEMENT

All animal experiments were conducted in accordance with the rules and regulations of the Animal Care and Use Committee of Jichi Medical University (Approval no. 22008‐02. Approval date 2022/12/21).

## Data Availability

Data and materials of this study are available from the corresponding author on reasonable request.
